# Simultaneous quantification of serum monounsaturated and polyunsaturated phosphatidylcholines as potential biomarkers for diagnosing non-small cell lung cancer

**DOI:** 10.1038/s41598-018-25552-z

**Published:** 2018-05-08

**Authors:** Yingrong Chen, Zhihong Ma, Jing Zhong, Liqin Li, Lishan Min, Limin Xu, Hongwei Li, Jianbin Zhang, Wei Wu, Licheng Dai

**Affiliations:** 1 Huzhou Key Laboratory of Molecular Medicine, Huzhou Central Hospital, Huzhou, 313000 P.R. China; 2 Cardiothoracic Surgery, Huzhou Central Hospital, Huzhou, 313000 P.R. China; 3 Internal Medicine, Huzhou Central Hospital, Huzhou, 313000 P.R. China

## Abstract

Non-small cell lung cancer (NSCLC) is one of the most common malignancies worldwide. In this study, we investigated Ultrahigh Performance Liquid Chromatography-Quadrupole Time-of-Flight Mass Spectrometry and Gas Chromatography Time-of-Flight/Mass Spectrometry-based non-targeted metabolomic profiles of serum samples obtained from early-stage NSCLC patients and healthy controls (HC). Metabolic pathways and the biological relevance of potential biomarkers were extensively studied to gain insights into dysregulated metabolism in NSCLC. The identified biomarker candidates were further externally validated via a targeted metabolomics analysis. The global metabolomics profiles could clearly distinguish NSCLC patients from HC. Phosphatidylcholine (PC) levels were found to be dysregulated in glycerophospholipid (GPL) metabolism, which was the top altered pathway in early-stage NSCLC. Compared with those in HC, significant increases in the levels of saturated and monounsaturated PCs such as PC (15:0/18:1), PC (18:0/16:0) and PC (18:0/20:1) were observed in NSCLC. Additionally, relative to those in HC, the levels of 9 polyunsaturated PCs, namely, PC (17:2/2:0), PC (18:4/3:0), and PC (15:0/18:2), and so on were significantly decreased in NSCLC patients. A panel of 12 altered PCs had good diagnostic performance in differentiating early-stage NSCLC patients from HC, and these PCs may thus be used as serum biomarkers for the early diagnosis of NSCLC.

## Introduction

Lung cancer is one of the most common malignancies worldwide, with the highest rate of morbidity and mortality. Non-small cell lung cancer (NSCLC) accounts for 85% of all lung cancer cases^1^. Despite significant advancements in the diagnosis and treatment of NSCLC, the five-year survival rate of NSCLC is a mere 15%. Unfortunately, 70% of NSCLC patients are diagnosed at advanced stages, which markedly reduce the effectiveness of treatment^2–4^. Early diagnosis plays a key role in patients’ prognoses. One of the key problems in the management of NSCLC is the lack of new molecular biomarkers with high sensitivity and specificity.

Metabolomics involves the analysis of all low molecular weight metabolites in a quantitative manner at a certain time under specific environmental conditions in an organism or a biological sample^5^. It is essential to distinguish between information pertaining to a diseased or non-diseased status. Metabolomics may have the potential for application in the field of disease diagnosis or in the identification of disease biomarkers^6–8^. NSCLC development and progression alter metabolic processes. Advancements are being made for a great diversity of technologies, including nuclear magnetic resonance (NMR) spectroscopy^9,10^, high performance liquid chromatography/mass spectrometry (HPLC/MS and LC/MS/MS)^11,12^, and gas chromatography/mass spectrometry (GC/MS)^13,14^. Metabolomics approaches include non-targeted metabolomics and targeted metabolomics. Non-targeted metabolomics approaches involve global profiling of the metabolome to identify different metabolites that can be used for the initial screening of diagnostic biomarkers. However, the accuracy and reliability of the identification of metabolites by these approaches are low^15,16^. Targeted metabolomics approaches use standards to quantify metabolites to validate biomarkers and investigate specific metabolic pathways identified using non-targeted metabolic profiling^17^. The accuracy and reproducibility are much higher for targeted metabolomics than for non-targeted metabolomics.—

To broaden our understanding of metabolic alterations in NSCLC and to identify potential biomarkers for early diagnosis, 90 early-stage NSCLC patients and 90 healthy controls (HC) were enrolled in a study involving Ultrahigh Performance Liquid Chromatography-Quadrupole Time-of-Flight Mass Spectrometry (UHPLC-Q-TOF/MS) and Gas Chromatography Time-of-Flight/Mass Spectrometry (GC-TOF/MS)-based non-targeted metabolomics analysis. Metabolic pathways and biological relevance of potential biomarkers were extensively studied to gain insights into dysregulated metabolism in early-stage NSCLC. The identified biomarker candidates were further externally validated in a cohort including 30 early-stage NSCLC patients and 30 HC by a targeted metabolomics analysis.

## Results

### Clinical characteristics of the study subjects

A total of 90 NSCLC patients (including 40 (44%) males and 50 (56%) females; mean age 58.1 ± 9.0 years) and 90 sex- and age-matched HC (42 males and 48 females; mean age 53.0 ± 11.8 years) were included in our non-targeted metabolomics study. For the targeted metabolomics study, 30 NSCLC patients and 30 HC were included in the absence of differences in characteristics such as age and sex between the two groups. The clinical characteristics of the subjects are summarized in Table 1.

**Table 1:** Clinical characteristics of early-stage NSCLC patients, healthy controls (HC) and patients with benign lung disease (LBD) enrolled in this study.

Characteristic	Non-targeted metabolomics		Targeted metabolomics	
	NSCLC patients	HC	NSCLC patients	HC
Sample size	90	90	30	30
Age range (years)	58.1 ± 9.0	53.0 ± 11.8	62.1 ± 6.7	51.7 ± 7.1
Sex				
Male	40	42	21	19
Female	50	48	9	11
Pathological type				
ADC^a^	50	—	15	—
SqCC^b^	30	—	15	—
TNM stage^c^				
Stage I	81	—	15	—
Stage II	9	—	15	—

^a^ADC: Adenocarcinoma.

^b^SqCC: Squamous cell carcinoma.

^c^Union for International Cancer Control (UICC) TNM Classification of lung cancer (8th ed., 2017).

### Non-targeted metabolomics analysis

#### Metabolic profiles of serum samples

**Figure 1:**
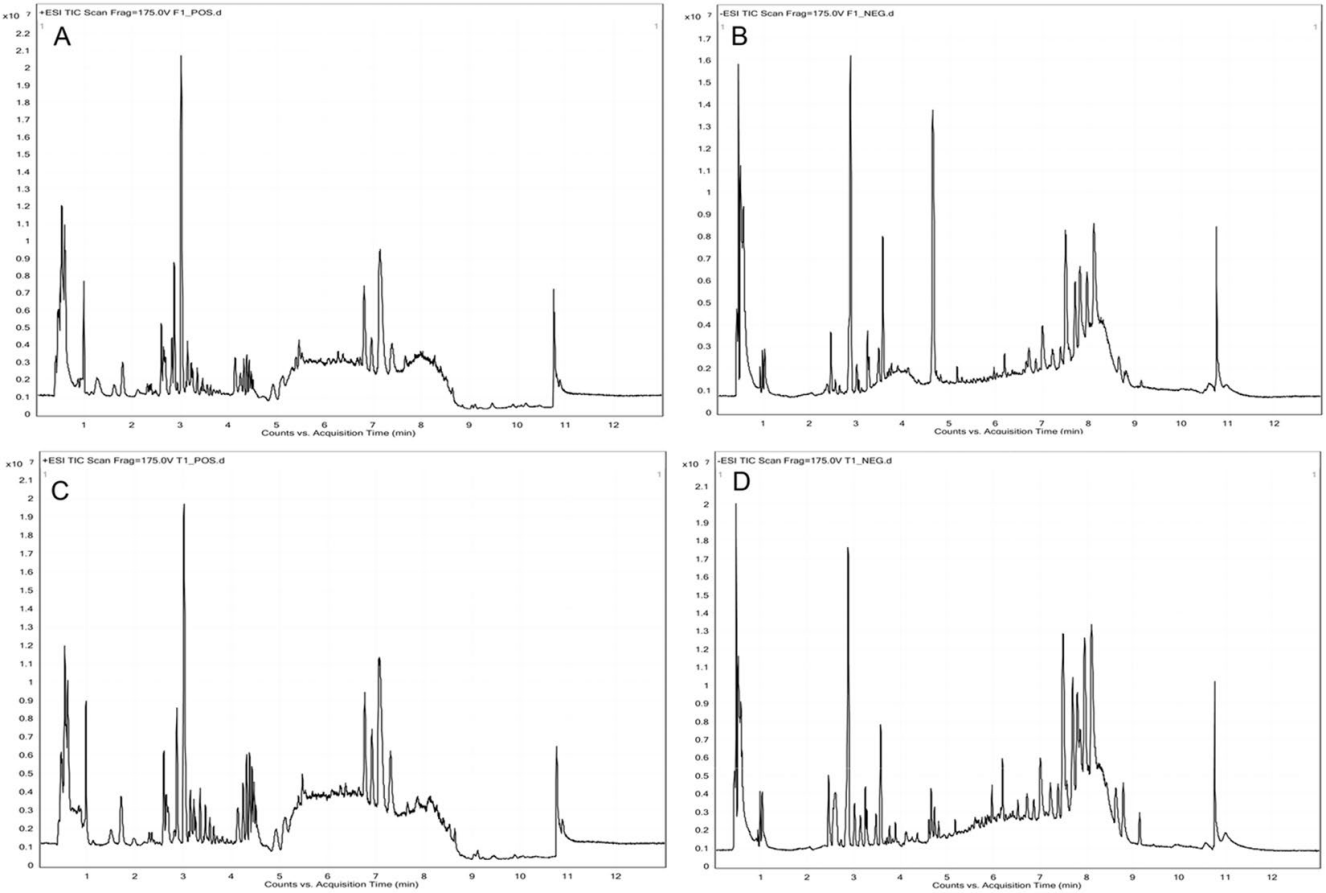
Typical TICs of metabolic profiles of early-stage NSCLC patients (**A**) positive ion mode; (**B**) negative ion mode and HC (**C**) positive ion mode; (**D**) negative ion mode based on UHPLC-Q-TOF/MS analysis.

**Figure 2:**
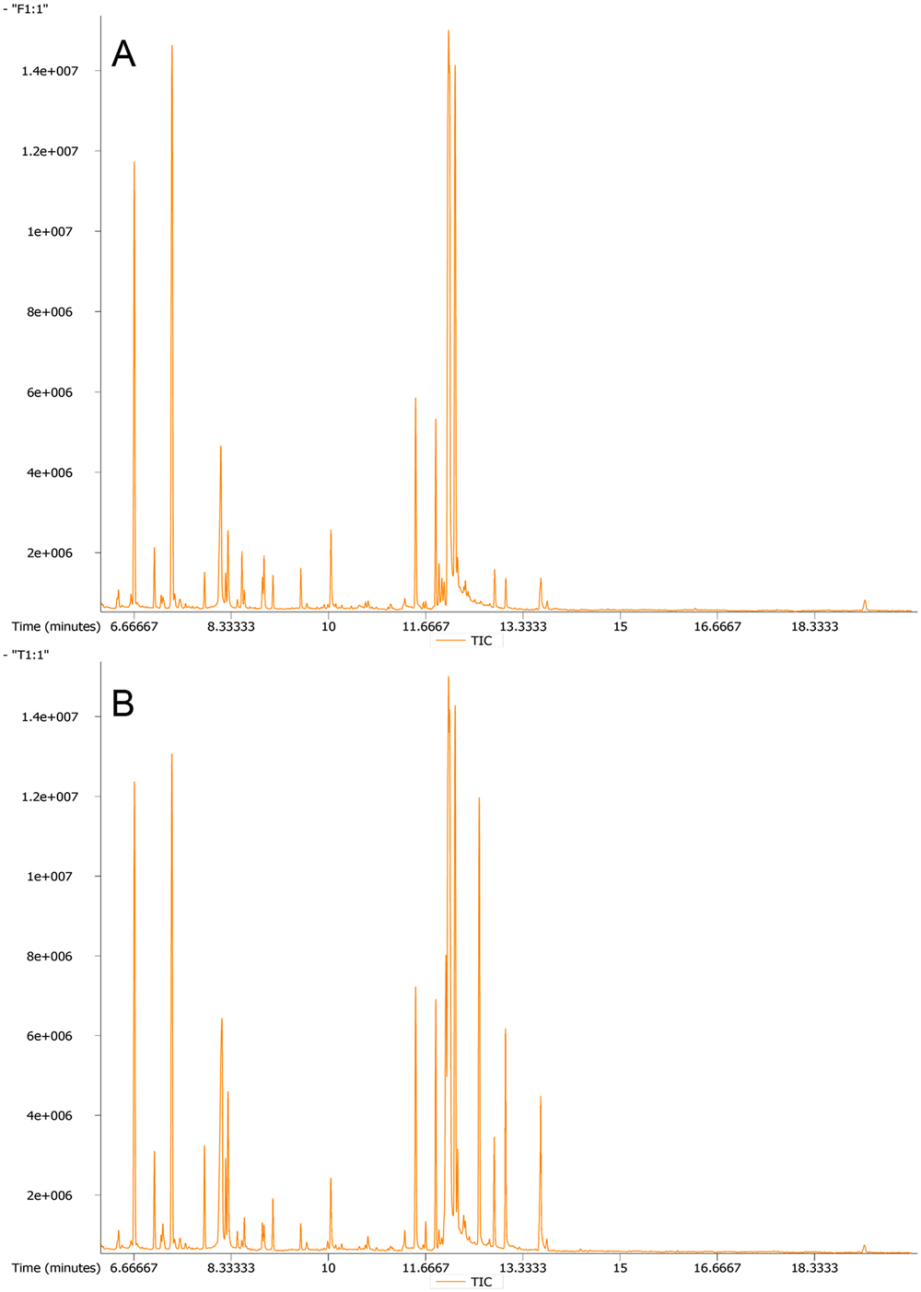
Typical TICs of metabolic profiles of early-stage NSCLC patients (**A**) and HC (**B**) based on GC-TOF/MS analysis.

For UHPLC-Q-TOF/MS, 1865 metabolite features in positive ion mode and 359 metabolite features in negative ion mode were selected for subsequent analyses. For GC-TOF/MS, 290 ion peaks were identified, and 223 metabolites remained after the removal of noise based on the interquartile range. Typical total ion chromatograms (TICs) of the metabolic profiles of early-stage NSCLC patients and HC are provided in Figs 1 and 2. The data were normally distributed after normalization.

#### Multivariate statistical analysis

**Figure 3:**
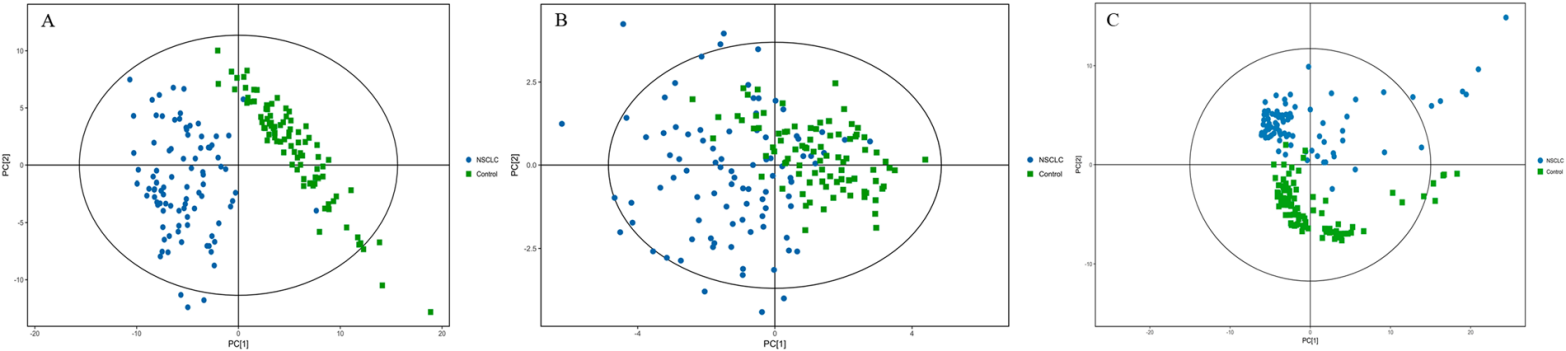
PCA score plots of metabolic profiles in early-stage NSCLC patients and HC with or without mean-centred (ctr) scaling. (**A**) UHPLC-Q-TOF/MS analysis in the positive mode, R^2^X = 0.627. (**B**) UHPLC-Q-TOF/MS analysis in the negative mode, R^2^X = 0.529. (**C**) GC-TOF/MS analysis, R^2^X = 0.416. Dots and boxes denote samples from early-stage NSCLC patient and HC, respectively.

**Figure 4:**
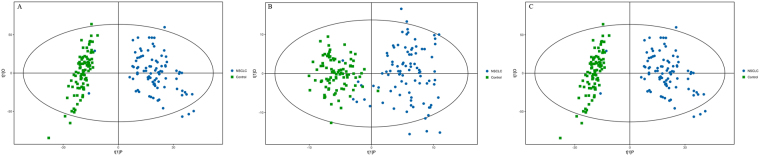
OPLS-DA score plot of metabolic profiles of early-stage NSCLC patients and HC after unit variance (uv) scaling. (**A**) UHPLC-Q-TOF/MS analysis in the positive mode. (**B**) UHPLC-Q-TOF/MS analysis in the negative mode. (**C**) GC-TOF/MS analysis.

The principle component analysis (PCA) score plots obtained for early-stage NSCLC patients and HC are shown in Fig. 3. The samples in the two groups segregated into two distinct clusters. A supervised orthogonal partial least squares discriminant analysis (OPLS-DA) was employed to maximize the differences between groups and to aid in the identification of marker metabolites responsible for class separation. The parameters of the OPLS-DA score plots (Fig. 4) were R^2^X = 0.637, R^2^Y = 0.866, and Q^2^ = 0.866 for UHPLC-Q-TOF/MS in the positive mode, R^2^X = 0.268, R^2^Y = 0.804, and Q^2^ = 0.760 for UHPLC-Q-TOF/MS in the negative mode and R^2^X = 0.134, R^2^Y = 0.905, and Q^2^ = 0.860 for GC-TOF/MS. These values indicated that there was a clear separation between early-stage NSCLC patients and HC.

#### Differences in metabolites and related pathways between NSCLC patients and HC

**Figure 5:**
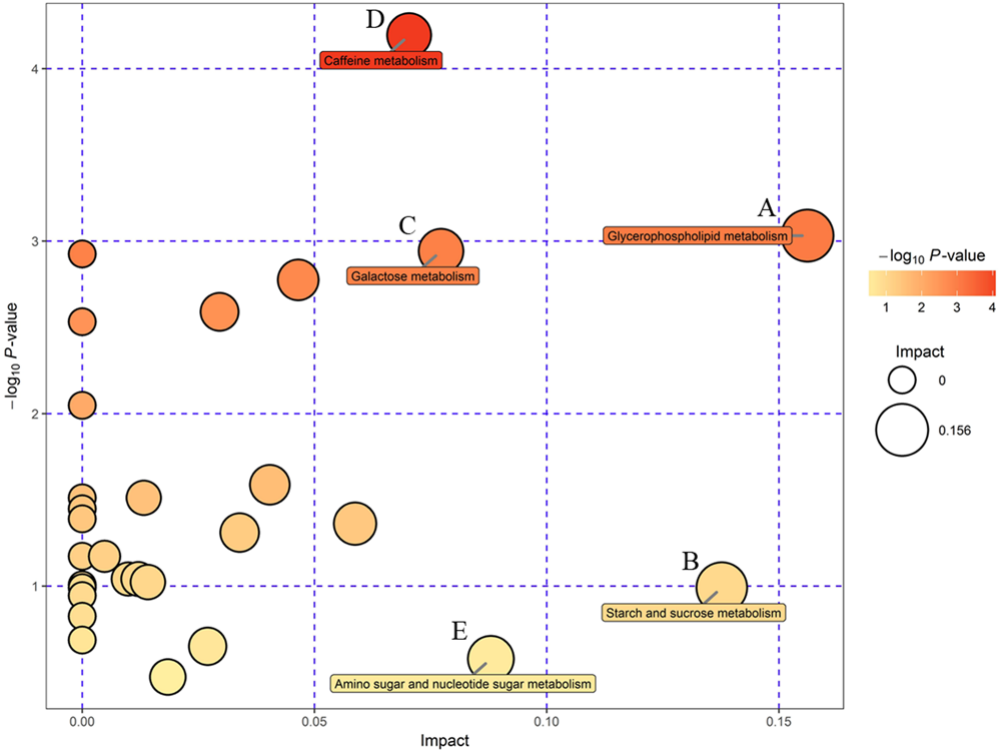
Summary of pathway analyses based on metabolomics data. (**A**) Glycerophospholipid metabolism. (**B**) Starch and sucrose metabolism. (**C**) Galactose metabolism. (**D**) Caffeine metabolism. (**E**) Amino sugar and nucleotide sugar metabolism.

**Figure 6:**
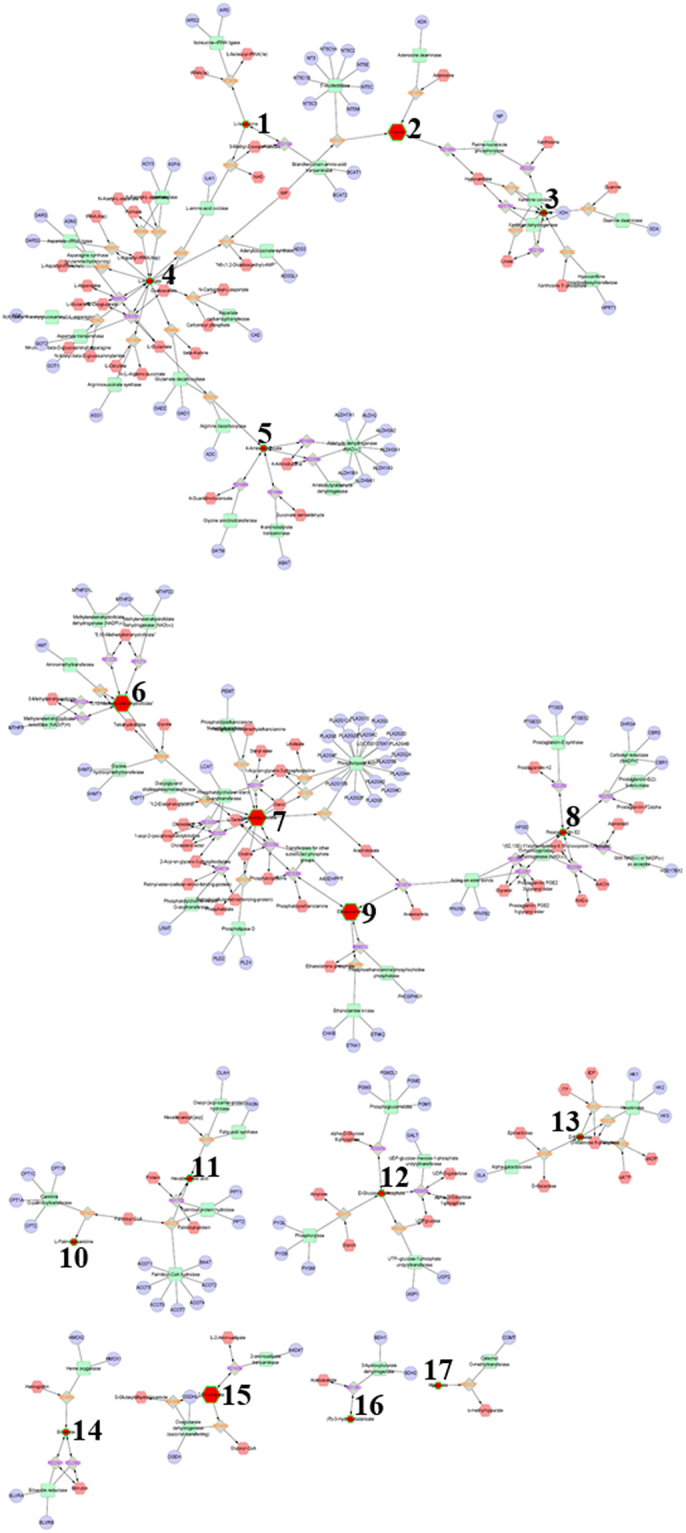
Networks of indicated differential metabolites. 1. L-Isoleucine. 2. Inosine. 3. Xanthine. 4. L-Aspartate. 5. 4-Aminobutanoate. 6. 5, 10-Methylenetrahydrofolate. 7. Phosphatidylcholine. 8. Prostaglandin E2. 9. Ethanolamine. 10. L-Palmitoylcarnitine. 11. Hexadecanoic acid. 12. D-Glucose-1-phosphate. 13. D-Mannose. 14. Biliverdin. 15. 2-Oxoadipate. 16. (R)-3-Hydroxybutanoate. 17. Hippurate.

For UHPLC-Q-TOF/MS, a total of 37 different metabolites were selected based on variable importance in the projection (VIP) values greater than 1, *p* values less than 0.05, and the *q* values of false discovery rate (FDR) less than 0.05 including 20 metabolites in the positive mode and 17 metabolites in the negative mode (Table 2). For GC-TOF/MS, 19 different metabolites were identified (Table 3). Then, we mapped these different metabolites into their biochemical pathways through metabolic enrichment and pathway analyses based on the KEGG database and MetaboAnalyst. As shown in Fig. 5, the significantly altered pathways were glycerophospholipid (GPL) metabolism, starch and sucrose metabolism, galactose metabolism, caffeine metabolism, and amino sugar and nucleotide sugar metabolism based on the results of UHPLC-Q-TOF/MS and GC-TOF/MS analyses. GPL metabolism was the top altered pathway in early-stage NSCLC. Next, networks of metabolites were constructed with Metscape (Fig. 6). Table 4 lists the detailed results of pathway analyses. The metabolites involved in the altered pathways included phosphatidylcholines (PCs), ethanolamine, glucose-1-phosphate, D-galactonate, theophylline and xanthine. The sensitivity, specificity and area under the ROC curve (AUC) of each metabolite and the combination of PCs are presented in Table 5. As shown in Table 5, the combination of PCs had high performance in predicting early-stage NSCLC with an AUC of 0.996. Thus, we selected PCs for the target metabolomics analysis.

**Table 2:** Discrepant metabolites identified by UHPLC-Q-TOF/MS analysis between early-stage NSCLC patients and HC.

Peak	Metabolite	Polarity	m/z	VIP	Fold-change	Peak	Metabolite	Polarity	m/z	VIP	Fold-change
1	5,10-Methylenetrahydrofolate	POS	440.1747	1.11	3.80	21	1-Methylxanthine	NEG	203.0023	1.01	0.63
2	Biliverdin	POS	583.2549	1.11	0.29	22	2-Hydroxy-3-methylbutyric acid	NEG	117.0554	1.09	1.39
3	β-Octylglucoside	POS	310.2229	1.73	0.39	23	2-Oxoadipic acid	NEG	141.0165	1.79	1.18
4	Glycochenodeoxycholate	POS	450.3173	1.07	1.28	24	3-Hydroxycapric acid	NEG	169.1228	1.54	0.50
5	Isovaleric acid	POS	103.0755	1.60	0.35	25	all cis-(6,9,12)-Linolenic acid	NEG	277.2175	1.25	0.80
6	L-palmitoylcarnitine	POS	444.3168	1.62	0.50	26	Arachidonic Acid	NEG	303.2327	1.26	0.84
7	PC(10:0/22:2)	POS	730.5311	1.49	0.36	27	DL-lactate	NEG	89.0240	2.41	2.03
8	PC(15:1/22:6)	POS	790.5513	1.54	1.57	28	Hippuric acid	NEG	178.0510	1.22	0.41
9	PC(16:1/0:0)	POS	494.3326	1.08	1.62	29	Inosine	NEG	267.0736	1.72	4.17
10	PC(17:2/24:4)	POS	848.5930	1.35	1.39	30	PA(18:2/18:0)	NEG	699.4770	1.81	1.21
11	PC(18:0/22:6)	POS	834.5778	1.57	1.64	31	Palmitic acid	NEG	255.2322	1.04	0.86
12	PC(18:3/0:0)	POS	518.3369	1.57	2.11	32	PE(16:0/0:0)	NEG	452.2774	1.35	1.48
13	PC(18:4/22:6)	POS	826.5287	1.03	1.69	33	PE(18:0/0:0)	NEG	480.3087	1.04	1.34
14	PC(19:0/0:0)	POS	538.3695	1.34	1.45	34	PE(18:1/0:0)	NEG	478.2930	1.11	1.67
15	PC(20:0/0:0)	POS	552.3926	1.48	0.51	35	PE(18:2/0:0)	NEG	476.2775	1.26	1.57
16	PC(O-16:0/0:0)	POS	504.3452	1.29	1.83	36	Theophylline	NEG	179.0544	1.19	0.42
17	PC(O-16:1/0:0)	POS	502.3333	1.34	1.49	37	Xanthine	NEG	188.9864	1.29	0.52
18	PC(O-18:3/0:0)	POS	504.3374	1.62	0.37						
19	PE(22:2/12:0)	POS	716.5143	1.15	1.29						
20	L-prolyl-L-phenylalanine	POS	263.1366	1.67	1.50

**Table 3:** Discrepant metabolites identified by GC-TOF/MS analysis between early-stage NSCLC patients and HC.

Peak	Similarity	Metabolite	VIP	Fold-change
1	326	3-(2-Hydroxyphenyl)propionic acid	1.50	0.81
2	279	4-Acetylbutyric acid 2	1.56	1.32
3	291	Galactonic acid	4.26	6.64
4	523	Ethanolamine	1.32	0.88
5	326	4-Androsten-19-ol-3,17-dione 1	3.67	11.59
6	789	2-Hydroxypyridine	1.10	0.83
7	814	Mannose 2	1.59	1.74
8	378	2-Deoxyerythritol	1.30	0.83
9	582	Sedoheptulose	1.28	0.77
10	780	Isoleucine	1.37	0.74
11	783	D-(glycerol-1-phosphate)	2.91	1.90
12	380	3-Hydroxybutyric acid	3.29	1.60
13	777	Aspartic acid 1	1.21	0.76
14	649	β-Glycerophosphoric acid	1.39	0.87
15	264	Prostaglandin E2 2	1.06	1.68
16	607	Glucose-1-phosphate	1.52	0.82
17	410	P-cresol	1.17	0.60
18	676	4-Aminobutyric acid 3	1.24	0.67
19	363	Lyxose 1	1.45	0.85

**Table 4:** Detailed results of pathway analyses based on metabolomics data.

	Total	Hits	Hit name	Raw *p*	−log(p)	FDR	Impact
Glycerophospholipid metabolism	39	2	1. Phosphatidylcholine (PC) 2. Ethanolamine	0.048	3.032	0.908	0.156
Starch and sucrose metabolism	50	1	1. Glucose-1-phosphate	0.371	0.991	1.000	0.138
Galactose metabolism	41	2	1. Glucose-1-phosphate 2. D-Galactonate	0.053	2.942	0.908	0.077
Caffeine metabolism	21	2	1. Theophylline 2. Xanthine	0.015	4.194	0.908	0.070
Amino sugar and nucleotide sugar metabolism	88	1	1. Glucose-1-phosphate	0.561	0.578	1.000	0.088

**Table 5:** Prediction performance of potential metabolic biomarkers between early-stage NSCLC and HC.

Biomarker	AUC (95% CI)	Sensitivity (%)	Specificity (%)
Combined effects of PCs	0.996 (0.987–1.000)	98.9	98.9
Ethanolamine	0.692 (0.612–0.771)	91.1	56.7
D-galactonate	0.880 (0.830–0.929)	86.7	72.2
Glucose-1-phosphate	0.316 (0.238–0.394)	86.7	50.0
Theophylline	0.251 (0.181–0.322)	67.8	76.7
Xanthine	0.264 (0.191–0.337)	77.8	60.0

### Targeted metabolomics analysis

**Figure 7:**
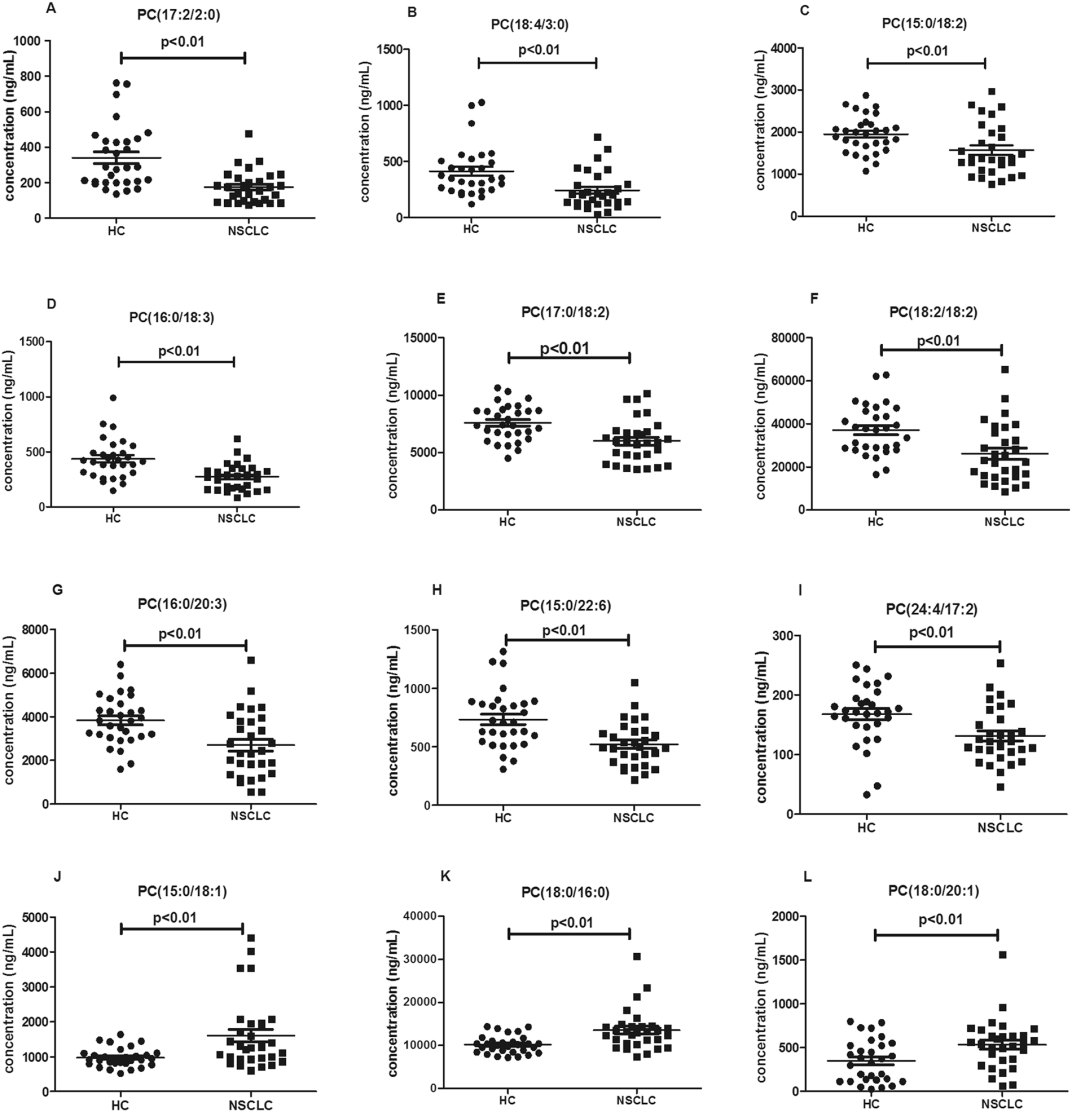
Scatter plots of serum levels of the selected PCs in HC and early-stage NSCLC patients. Black horizontal lines represent median values. P values are determined by the Student’s t-tests.

We analysed the changes in the concentration of 85 PCs in early-stage NSCLC patients and HC. The data were normally distributed after normalization. The *p* value based on Student’s t-test and the *q* values of FDR in the average concentration of PCs were calculated between NSCLC patients and HC (Table S1). Only 12 PCs were selected as biomarkers for the early diagnosis of NSCLC (Table 6) according to *p* < 0.01 and *q* < 0.05. The concentration distributions of these selected PCs are shown in Fig. 7. As shown in Fig. 7, compared with the corresponding levels in HC, the levels of saturated and monounsaturated PCs such as PC(15:0/18:1), PC(18:0/16:0) and PC(18:0/20:1) were significantly increased, whereas the levels of polyunsaturated PCs such as PC(17:2/2:0), PC(18:4/3:0), PC(15:0/18:2), PC(16:0/18:3), PC(17:0/18:2), PC(18:2/18:2), PC(16:0/20:3), PC(15:0/22:6) and PC(24:4/17:2) significantly decreased in early-stage NSCLC patients.

**Table 6:** Detection of PCs as potential biomarkers for the diagnosis of early-stage NSCLC.

No.	Biomarker	*p* value	*q* value	Fold-change	AUC (95% CI)	Sensitivity (%)	Specificity (%)	Trend (Cancer)
1	PC(17:2/2:0)	1.86E-05	6.38E-04	0.51	0.183 (0.078–0.288)	66.7	86.7	down
2	PC(18:4/3:0)	1.18E-03	6.07E-03	0.59	0.233 (0.110–0.356)	66.7	83.3	down
3	PC(15:0/18:2)	7.15E-03	1.92E-02	0.81	0.283 (0.147–0.420)	56.7	90.0	down
4	PC(16:0/18:3)	1.13E-03	2.27E-02	0.63	0.218 (0.101–0.335)	83.8	70.0	down
5	PC(17:0/18:2)	5.72E-04	4.36E-03	0.79	0.250 (0.125–0.375)	70.0	73.3	down
6	PC(18:2/18:2)	1.05E-03	5.81E-03	0.69	0.254 (0.128–0.381)	53.3	93.3	down
7	PC(16:0/20:3)	1.42E-03	6.48E-03	0.65	0.252 (0.126–0.378)	60.0	90.0	down
8	PC(15:0/22:6)	4.00E-04	3.63E-03	0.71	0.243 (0.121–0.365)	53.5	90.0	down
9	PC(24:4/17:2)	3.78E-03	1.30E-02	0.78	0.273 (0.139–0.407)	70.0	80.0	down
10	PC(15:0/18:1)	8.26E-03	2.07E-02	1.64	0.717 (0.586–0.848)	60.0	80.0	up
11	PC(18:0/16:0)	5.27E-03	1.62E-02	1.34	0.763 (0.641–0.885)	73.3	76.7	up
12	PC(18:0/20:1)	1.96E-03	3.28E-02	1.53	0.691 (0.556–0.826)	66.7	70.0	up
	Panel a	—		—	0.897 (0.818–0.975)	90.0	76.7	
	Panel b	—		—	0.811 (0.703–0.919)	76.7	70.0	
	Panel c	—		—	1.000 (1.000–1.000)	100	100	

Panel (a): combination of down-regulated PCs; Panel b: combination of up-regulated PCs.

Panel (c): combination of 12 altered PCs.

**Figure 8:**
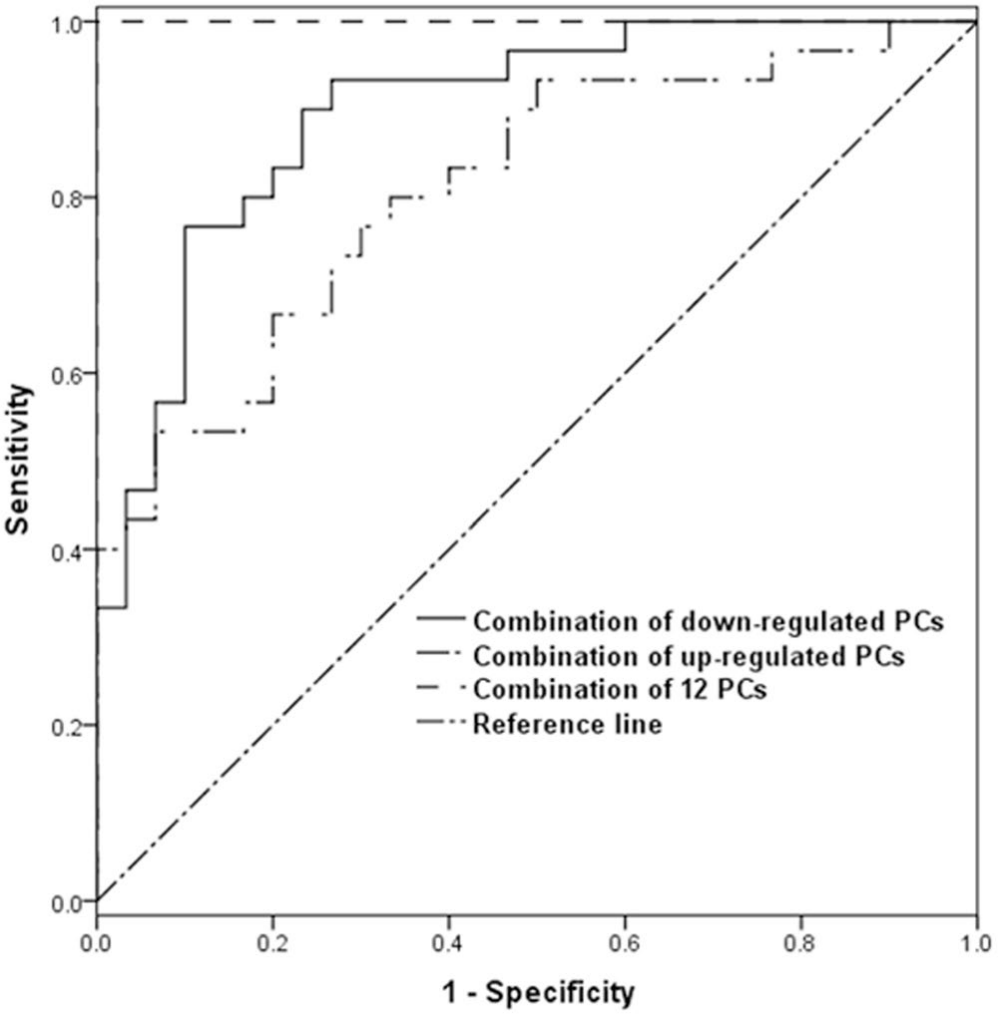
ROC curves of the combination of PCs that were altered between HC and early-stage NSCLC patients.

To estimate the diagnostic value of the twelve targeted PCs, we further performed receiver operating characteristic (ROC) analysis. The sensitivity, specificity and AUC of each metabolite and the combination of PCs are presented in Table 6. The ROC curves are shown in Fig. 8. Although individual PCs did not have good diagnostic performance in distinguishing NSCLC from HC, the combination of these twelve PCs had the best diagnostic performance.

## Discussion

In this study, we performed a comprehensive non-targeted metabolomics analysis in human serum samples to identify differences in metabolic features between HC and NSCLC patients by UHPLC-Q-TOF/MS and GC-TOF/MS. Metabolic pathway analysis of altered metabolites suggested that GPL metabolism was the most significantly altered metabolic pathway between the two groups. We then employed a targeted metabolomics analysis to further evaluate changes in the levels of PCs between early-stage NSCLC patients and HC. ROC analysis revealed that a panel of 12 PCs exhibited good performance in differentiating HC and early-stage NSCLC patients.

NSCLC is the most frequently diagnosed cancer with high mortality, partly ascribed to late diagnosis and poor prognosis. Many of the commonly used serum tumour biomarkers are limited to late-stage disease and have low sensitivity and specificity^18,19^. Currently, there are a handful of validated small molecular biomarkers for NSCLC that can be used to avoid the necessity of tumour biopsies for classifying NSCLC. Metabolomics is used to systematically analyse all metabolites and metabolic pathways in an organism or a biological sample and is being increasingly recognized as a technique enabling the discovery of biomarkers and understanding of disease mechanisms^20^. Non-targeted and targeted metabolomics are the two main MS techniques for the study of metabolites and have their own advantages and disadvantages. Non-targeted metabolomics is good for the initial screening of biomarkers but has low precision. In contrast, targeted metabolomics has high sensitivity and good reproducibility in validating biomarkers^21^. In the past, non-targeted metabolomics was widely applied for identifying NSCLC biomarkers by NMR, HPLC/MS and GC/MS. It has been reported that the major alterations in lung adenocarcinoma are related to phospholipid metabolism and protein catabolism, whereas squamous cell carcinoma exhibits greater changes in glycolytic and glutaminolytic pathways based on NMR^9^. In one study, GC-TOF/MS was used to measure altered metabolites in paired malignant and non-malignant lung tissues from early-stage adenocarcinoma, and cancer-associated biochemical alterations were characterized by decreased levels of glucose and elevated levels of cysteine, antioxidants, alpha- and gamma-tocopherol, and the nucleotide metabolite 5,6-dihydrouracil^22^. Metabolomic profiling by UPLC-Q-TOF/MS has been performed to identify diagnostic and prognostic markers in lung cancer. Creatine riboside, a novel molecule identified in this study, and N-acetylneuraminic acid were both significantly elevated in NSCLC and associated with poor prognosis. Creatine riboside was the strongest classifier of lung cancer status in the whole cohort as well as in stage I–II cases and thus may be important for early detection^23^. The different metabolites should be further validated by targeted metabolomics. Zhang *et al*. characterized metabolic alterations in lung cancer using a non-targeted metabolic profiling strategy based on 1H-NMR spectroscopy and a targeted metabolic profiling strategy based on rapid resolution liquid chromatography (RRLC). Sixteen altered metabolites were detected using the non-targeted approach, and nine were identified using the targeted approach^10^.

In our study, both UHPLC-Q-TOF/MS and GC-TOF/MS were simultaneously used for non-targeted metabolomics analysis to explore alterations in serum levels of metabolites between early-stage NSCLC patients and HC. For targeted metabolomics, UHPLC-Triple-TOF/MS was used to validate the concentration of PCs and demonstrated high performance in predicting early-stage NSCLC. Because the purpose of the non-targeted metabolomics analysis was to select different metabolites and the detection method used in non-targeted metabolomics was not specialized for PCs, the results needed to be verified using a specialized quantitative method for PCs. Thus, the targeted metabolomics analysis was used to determine the absolute concentrations of the PCs. Because of the different aims between the two methods, the methods for extracting metabolites from the sample were different, and the methods of the chromatography and mass spectrometry were also different. Although the untargeted metabolomics could detect some lipids, it was not a suitable method for the lipid determination. Because of the limitation of the untargeted metabolomics in the extracting and determining lipids, more PCs were extracted and identified by the targeted analysis. We suspected it was the reason that the significant PCs identified in the untargeted analysis were non-significant in the targeted analysis. However, we believe that the two methods are complementary and irreplaceable. On the other head, the targeted analysis was validated only in 30 early-stage NSCLC patients and 30 HC, which should be verified in larger samples in the future to increase their credibility.

In this study, GPL metabolism was the top altered pathway in the NSCLC samples, and serum concentrations of PCs were altered between early-stage NSCLC patients and HC. Phospholipids, one of the major components of cell membranes, participates in various biological functions, and their levels are altered in various human cancers^24,25^. PCs are the most abundant bilayer-forming phospholipids in eukaryotic membranes and can contribute to proliferative growth in cancer cells^26,27^. Abnormal PC metabolism has been reported in cancer cells. Guo *et al*. reported that different combinations of sphingomyelin (SM) (34:1), PC(34:2), PC(34:1), PC(36:4), PC(36:3), and PC(36:2) exhibit high diagnostic potential for lung cancer, colorectal cancer, gastric cancer and pancreatic cancer^28^. Increased PCs levels have also been reported in cervical cancer, ovarian cancer, breast cancer and oesophageal squamous cell carcinoma and thus might be interpreted as a requirement for the high rate of cancer cell proliferation. Additionally, increased levels of PCs may be correlated with the overexpression of choline kinase in various cancers^29^. In our study, the levels of monounsaturated phospholipids, such as PC(15:0/18:1) and PC(18:0/20:1), were significantly increased, whereas the levels of polyunsaturated phospholipids such as PC(17:2/2:0), PC(18:4/3:0), PC(15:0/18:2), PC(16:0/18:3), PC(17:0/18:2), PC(18:2/18:2), PC(16:0/20:3), PC(15:0/22:6) and PC(24:4/17:2) were significantly decreased in NSCLC samples. Previous studies have noted that the activation of de novo lipogenesis is an early and common event in the tumour microenvironment^30–32^. The key enzymes in this pathway include fatty acid synthase and stearoyl-CoA desaturase-1. Guo *et al*.^29^ reported the up-regulation of fatty acid synthase and stearoyl-CoA desaturase-1 in the lung cancer microenvironment relative to that in adjacent normal tissues. Thus, we speculated that the promotion of biosynthesis of lipids with monounsaturated acyl chains and the suppression of biosynthesis of polyunsaturated lipids in the NSCLC microenvironment may be activated by de novo lipogenesis. Increased lipid saturation can help reduce cell membrane fluidity and promote tumour cell invasion and infiltration^32,33^. Ollila S *et al*.^33^ reported on the influence of unsaturated lipids on single-component membrane properties, focusing on their dynamic aspects and lateral pressure profiles across the membrane. With increasing degree of unsaturation, the authors observed considerable effects on dynamic properties, such as accelerated dynamics of phosphocholine head groups and glycerol backbones as well as accelerated rotational dynamics of lipid molecules. The lateral pressure profile is also altered by the degree of unsaturation. With increasing numbers of double bonds, the peak in the middle of the bilayer decreases. This is compensated for by changes in the membrane-water interface region via increasing peak heights of the lateral pressure profile. Hilvo *et al*.^31^ reported that increased levels of saturated fatty acids, associated with reduced membrane fluidity, are also found in aggressive breast cancers, suggesting that reduced membrane fluidity is a feature of the advanced disease. Rysman *et al*.^32^ also reported that alterations in fatty acid saturation can dramatically alter these properties and affect many aspects of the cellular machinery. The shift from lipid uptake to de novo lipogenesis in cancer cells leads to increased membrane lipid saturation, resulting in higher levels of saturated and monounsaturated phospholipids, potentially protecting cancer cells from oxidative damage by reducing lipid peroxidation. These findings underline the importance of precisely controlled regulation of lipid synthesis and desaturation in cancer cells. Thus, we speculated that the de novo lipogenesis pathway may promote the synthesis of monounsaturated PC molecules to enhance cell membrane formation and increase plasma membrane density, thereby altering cell membrane fluidity and promoting tumour formation in NSCLC.

In summary, we observed a significantly altered metabolic profile in early-stage NSCLC using UHPLC-Q-TOF/MS- and GC-TOF/MS-based non-targeted metabolomics analysis and identified a panel of PCs to distinguish HC and NSCLC patients. The identified PCs were further externally validated by a targeted metabolomics analysis. Increased saturated and monounsaturated PCs and decreased polyunsaturated PCs may be used as potential biomarkers to differentiate early-stage NSCLC patients and HC. Our study has thus highlighted the power of using comprehensive metabolomics approaches to identify biomarkers and underlying mechanisms in NSCLC.

## Materials and Methods

### Chemicals

LC grade acetonitrile (ACN), methanol (MeOH), MTBE and dichloromethane were purchased from Merck (Darmstadt, Germany). L-2-chlorophenylalanine was purchased from Shanghai Heng Bo Biological Technology Co., Ltd. (Shanghai, China). BSTFA (1% TMCS, v/v) was purchased from REGIS Technologies. Inc. (Illinois, USA). Ultrapure water was prepared using a Milli-Q system (Millipore; Billerica, MA, USA). Lipidomix Mass Spec Standard (Catalogue No. 330707, containing 160 μg/mL PC(15:0/18:1) (d7)) was purchased from Avanti Polar lipids (Alabaster, AL, USA).

### Patients and sample collection

Serum samples were collected from NSCLC patients and HC at Huzhou Central Hospital from January 2015 to July 2016. The patients were selected according to the following criteria: (1) all patients were diagnosed and confirmed by pathology; (2) patients with NSCLC were at early stages (Stage I and II) according the clinical staging method and had no other cancers; and (3) none of the patients received preoperative adjuvant chemotherapy or radiotherapy. Serum samples from HC were collected from healthy volunteers with no history of carcinoma. Histopathology results for all cancer patients were confirmed by surgical resection of the tumours, while clinicohistopathological characteristics and tumour stages were assessed based on biopsy results. No preoperative chemotherapy or radiotherapy was administered to the cancer patients included in this study.

All samples were collected in accordance with ethical guidelines, and written informed consent was received. All patients were approached based on approved ethical guidelines, and patients who agreed to participate in this study were required to sign consent forms before being included in the study. The study was approved by Research Ethics Committee of Huzhou Centre Hospital. (Huzhou City, Zhejiang Province). We also confirmed that all methods were performed in accordance with the relevant guidelines and regulations.

Before the collection of serum samples, patients and healthy volunteers fasted for at least 12 h. Briefly, for serum isolation, blood was collected into tubes (BD Vacutainer with increased silica act clot activator and silicone-coated interior) and centrifuged at 700 g for 10 min at 4 °C within 2 h of venipuncture. The supernatant was removed and centrifuged in the same way for the second time. The resultant serum was transferred into a clean tube and stored at −80 °C until use.

### Non-targeted metabolomics

#### Sample preparation

For UHPLC-Q-TOF/MS, 700 μL of MeOH and 40 μL of L-2-chlorophenylalanine (1 mg/mL stock in dH_2_O) were added to 200 μL of each serum sample, and the samples were vigorously vortexed for 30 s. The mixtures were sonicated for 10 min (in ice water) and allowed to stand for 2 h at −20 °C. The solutions were centrifuged at 13,000 rpm for 15 min at 4 °C. A 400 μL aliquot of the supernatant was subjected to UHPLC-Q-TOF/MS. Additionally, 10 μL of each sample was taken and pooled as quality control (QC) samples.

For GC-TOF/MS, 700 μL of MeOH and 40 μL of L-2-chlorophenylalanine (1 mg/mL stock in dH_2_O) were added to 200 μL of each serum sample, and the samples were vigorously vortexed for 10 s. The samples were centrifuged at 13,000 rpm for 15 min at 4 °C, and a 300-μL aliquot of the supernatant was transferred to a clean vial while 10 μL of each sample was taken and pooled as QC samples. After being dried in a vacuum concentrator, the collected supernatant was suspended in 40 μL of methoxy pyridine hydrochloride (20 mg/mL in pyridine) and incubated for 30 min at 80 °C. After this incubation, 60 μL BSTFA with 1% TMCS was added to each vial, and the mixtures were incubated for 2 h at 70 °C. Then, 10 μL of a standard mixture of fatty acid methyl esters (FAMEs, 1 mg/mL C8-C16 and 0.5 mg/mL C18-C30 in chloroform) was added to the QC sample. After completion of the reaction, samples were prepared for GC-TOF/MS.

#### Chromatography and mass spectrometry

UHPLC-Q-TOF/MS analyses were performed using a UHPLC system (1290 series, Agilent Technologies, USA) coupled to a Q-TOF mass spectrometer (Agilent 6550 iFunnel Q-TOF, Agilent Technologies, USA). Waters ACQUITY UHPLC HSS T3 C18 column (1.7 μm, 2.1 mm × 100 mm) was used for LC. The column was maintained at 25 °C. The injected sample volume was 1 μL for the positive mode and 3 μL for the negative mode. The gradient conditions were as follows: 0–1 min in 1% B, 1–8 min linear gradient from 1 to 100% B, and 8–10 min in 100% B. Solvent A was 0.1% formic acid (FA) in water in the positive mode or 0.5 mmol/L NH_4_F in water in the negative mode, and solvent B was 0.1% FA in ACN in the positive mode or 100% ACN in the negative mode. The flow rate was 500 μL/min. The measurement conditions of MS data acquisition were as follows: drying gas temperature, 250 °C; drying gas flow, 16 L/min; nebulizer pressure, 20 psi in the positive mode or 40 psi in the negative mode; sheath gas temperature, 400 °C; sheath gas flow, 12 L/min; capillary voltage, 3,000 V in the positive mode or −3,000 V in the negative mode; and nozzle voltage, 0 V. The acquisition rate was set as 4 Hz, and the scan range was from a mass-to-charge ratio (m/z) of 50 to 1200 Da. Data acquisition for tandem mass spectrometry (MS/MS) was performed using another Q-TOF mass spectrometer (Triple TOF 6600, AB SCIEX, USA). QC samples were used for MS/MS data acquisition. The source parameters were set as follows: GAS1, 40 psi; GAS2, 80 psi; curtain gas pressure (CUR), 25 psi; heater gas temperature (TEM), 650 °C; ISVF, 5500 V in the positive mode and −4500 V in the negative mode; DP, 60 V; and CE, 35 ± 15 eV. To expand the coverage of the MS/MS spectra, the mass ranges were divided into the following four segments: 50–300 Da, 290–600 Da, 590–900 Da, and 890–1200 Da. Data acquisition and processing were performed with Mass Hunter (version B.05.01, Agilent Technologies, USA) and Analyst TF (version 1.7.1, AB SCIEX, USA) qualitative analysis software. MS raw data files were converted to the mzXML format using ProteoWizard and processed by the R package XCMS (version 1.41.0), which can perform peak finding, filtering, alignment, and scaling. The R package CAMERA was used for peak annotation after XCMS data processing^34^. The cutoff for match score was set as 0.1, and the minfrac was set as 0.5. All the m/z errors were less than 30 ppm, and all the retention time (RT) errors were less than 60 s. Metabolite identification was performed by matching the acquired MS/MS data against MS/MS data in a database developed in-house.

GC-TOF/MS analyses were performed using an Agilent 7890B GC system (Agilent, USA) coupled with a Pegasus HT TOF mass spectrometer (LECO, St. Joseph, MI, USA). The system was equipped with a DB-5MS capillary column coated with 5% diphenyl cross-linked with 95% dimethylpolysiloxane (30 m × 250 μm inner diameter, 0.25 μm film thickness; J&W Scientific, Folsom, CA, USA). A 1 μL aliquot of derivatized sample was injected in the splitless mode. Helium was used as carrier gas at a constant flow rate of 1 mL/min through the column. The front inlet purge flow was 3 mL/min. The column temperature was initially at 50 °C; after 1 min, the temperature was increased from 50 to 310 °C at a rate of 20 °C/min and held at 310 °C for 6 min. The injection, transfer line, and ion source temperatures were 280, 270, and 220 °C, respectively. The energy was −70 eV in electron impact mode. The MS data were acquired in full-scan mode with an m/z range of 50–500 at a rate of 20 spectra/s after a solvent delay of 366 s. Chroma TOF 4.3X software (LECO) and LECO-Fiehn Rtx 5 database were used for extracting raw peaks, filtering and calibrating data baselines, aligning peaks, performing deconvolution analysis, identifying peaks and integrating peak areas. The RT index (RI) method was used for peak identification, and the RI tolerance was 5,000. Metabolic features detected in less than 50% of all QC samples were discarded^35^. The UHPLC-Q-TOF/MS and GC-TOF/MS data were normalised and evaluated the distribution by MetaboAnalystR.

#### Multivariate statistical analysis

SIMCA-P 14.1 (Umetrics, Umea, Sweden) was employed for multivariate analysis, including PCA with mean-centred (ctr) scaling and OPLS-DA with unit variance (uv) scaling. A sevenfold cross-validation method was used based on the OPLS-DA model to estimate the robustness and the predictive ability of our model.

#### Selection of potential metabolic biomarkers for targeted metabolomics

Potential metabolic biomarkers were selected with VIP values greater than 1, *p* values based on Student’s t-test less than 0.05 and *q* values of FDR less than 0.05. In addition, the differentially abundant metabolites were cross-referenced to pathways by further searching commercial databases, including KEGG (http://www.genome.jp/kegg/) and MetaboAnalyst. Network of metabolites was built and analysed in Metscape, which is a plugin for Cytoscape.

Then, we constructed univariate and multivariate ROC curves for each biomarker and the combination of potential serum biomarkers to examine their utility for predicting early-stage NSCLC. The sensitivity and specificity trade-offs were summarized for each variable using the AUC. An AUC value of 1.0 corresponds to a prediction model with 100% sensitivity and 100% specificity, whereas an AUC value of 0.5 corresponds to a poor predictive model.

### Targeted metabolomics analysis

#### Sample preparation

Forty microlitres of each sample was added to 160 μL of water and 480 μL of extraction liquid (V_MTBE_:V_MeOH_ = 5:1, containing 10 μL of 160 μg/mL PC(15:0/18:0)(d7)) and vigorously vortexed for 60 s. The mixtures were sonicated for 10 min and centrifuged at 3,000 rpm for 15 min at 4 °C. A 200 μL aliquot of the supernatant was taken. The remaining lower fraction was mixed with 200 μL of MTBE and vigorously vortexed for 60 s. The mixtures were sonicated for 15 min centrifuged at 3,000 rpm for 15 min at 4 °C. A 200 μL aliquot of the supernatant was taken, and sonication, centrifugation and supernatant collection were repeated once more. The three supernatants (MTBE extracts) were transferred to a clean vial and dried in a vacuum concentrator. The dried samples were reconstituted with 80 μL of dichloromethane/MeOH (1:1, v/v) and subjected to UHPLC-MS/MS analysis. Additionally, 6 μL of each sample was taken and pooled as QC samples.

#### Chromatography and mass spectrometry

Lipid profiling was performed with a UHPLC system (1290 series, Agilent Technologies, USA) coupled with a Q-TOF mass spectrometer (Triple TOF 6600, AB SCIEX, USA). Phenomenex Kinetex C18 100 A column (1.7 μm, 2.1 × 100 mm) (Phenomenex, USA) was used for the separation of lipid extracts. The column was maintained at 25 °C. The linear gradient started from 60% to 0% A (10 mmol/L ammonium formate, ACN:H_2_O = 6:4) and 40% B (10 mmol/L ammonium formate, IPA:H_2_O = 9:1). Gradient conditions were as follows: 0–12 min linear gradient from 40 to 100% B and 12–13.5 min in 100% B. The flow rate was 300 μL/min. The injected sample volume was 1 μL. Data acquisition and processing were performed with the acquisition software Analyst TF (version 1.7.1, AB SCIEX, USA), which can simultaneously acquire high resolution MS and MS/MS data by full-scan TOF MS and information-dependent acquisition (IDA) in both positive and negative ion modes. The source parameters were set as follows: GAS1, 60 psi; GAS2, 60 psi; CUR, 30 psi; TEM, 600 °C; ISVF, −4500 V in the negative mode; and CE, 45 ± 25 eV.

#### Data processing

The data were processed using an absolute quantitative lipidomics method^36^. MS raw data files were converted to the mzXML format using MSConverter, and processed by the R package XCMS (version 1.41.0). The pre-processed results generated a data matrix that consisted of the RT, m/z, and peak intensity. The cutoff for match score was set as 0.8, and the minfrac was set as 0.5. All m/z errors are less than 30 ppm, and all RT errors are less than 60 s. The metabolic features detected in less than 50% of QC samples were discarded^35^. Lipid identification was performed by matching the acquired MS/MS data against MS/MS data in a database developed in-house. The absolute concentration (ng/mL) of each PC was calculated based on the peak area of the PC identified in the sample and the peak area of the internal standard of PC (15:0/18:1) corresponding to the sample (the formula was shown in Figure S1).

#### Statistics analysis

The data were normalised and evaluated the distribution by MetaboAnalystR. SPSS 19.0 software was used for statistical analyses. Data were presented as the mean ± SD. Differences between the two groups were evaluated by Student’s t-tests. Differences were considered statistically significant when *p* values were less than 0.01 and *q* values of FDR less than 0.05.

### Data availability

The datasets generated and/or analysed during the current study are available from the corresponding author upon request.

## Electronic supplementary material

supplementary file
