# Supplementary material for: Simultaneous quantification of serum monounsaturated and polyunsaturated phosphatidylcholines as potential biomarkers for diagnosing non-small cell lung cancer
Source: Sci Rep. 2018 May 8;8:7137. doi: 10.1038/s41598-018-25552-z (PMC5940703; doi:10.1038/s41598-018-25552-z)
Supplement: Supplementary file 1 — supplementary file [file 41598_2018_25552_MOESM1_ESM.docx]

Simultaneous quantification of serum monounsaturated and polyunsaturated phosphatidylcholines as potential biomarkers for diagnosing non-small cell lung cancer

Yingrong Chen^1^, Zhihong Ma^1^, Jing Zhong^1^, Liqin Li^1^, Lishan Min^1^, Limin Xu^1^, Hongwei Li^2^, Jianbin Zhang^2^, Wei Wu^3^ & Licheng Dai^1^

^1^Huzhou Key Laboratory of Molecular Medicine, Huzhou Central Hospital, Huzhou, 313000, P.R. China.

^2^Cardiothoracic Surgery, Huzhou Central Hospital, Huzhou, 313000, P.R. China.

^3^Internal Medicine, Huzhou Central Hospital, Huzhou, 313000, P.R. China.

Address correspondence to L.D. (e-mail: dlc171@hzhospital.com)

**Strategy of PCs quantification method**

First, a known concentration of PC (160 μg/mL PC (15:0/18:0) (d7) as SIL-IS) was added to the samples.

Second, the peak areas of the internal standard and the PCs in the samples were determined.

Third, use the RF of PCs provided in the reference (RF=1.51)^1^.

At last, substitute the above data into the formula (Figure S1), then the absolute concentrations of the PCs were calculated.

**References**

1. Tu, J., *et al.* Absolute quantitative lipidomics reveals lipidome-wide alterations in aging brain. *Metabolomics* 14: 5 (2018).

**Table S1.** The average concentration of PCs by targeted metabolomics in NSCLC patients and HC

| Peak | Metabolite | m/z | Rt(min) | Concentration (ng/mL) | | *p* | *q* |
| --- | --- | --- | --- | --- | --- | --- | --- |
|  |  |  |  | NSCLC | HC |  |  |
| 1 | PC(17:2/2:0) | 592.3214 | 74.36 | 163.47 | 340.06 | 1.86E-05 | 6.38E-04 |
| 2 | PC(15:1/4:0) | 594.3375 | 89.86 | 71.83 | 101.28 | 1.28E-02 | 2.52E-02 |
| 3 | PC(18:4/3:0) | 616.3219 | 72.48 | 235.84 | 411.84 | 1.18E-03 | 6.07E-03 |
| 4 | PC(22:5/7:0) | 726.4513 | 97.11 | 246.13 | 263.28 | 0.65 | 0.39 |
| 5 | PC(16:0/14:1) | 748.5089 | 277.85 | 275.77 | 172.01 | 5.33E-02 | 6.26E-02 |
| 6 | PC(16:0/14:0) | 750.5466 | 268.59 | 34.86 | 41.39 | 3.75E-02 | 4.94E-02 |
| 7 | PC(20:3/12:0) | 772.5085 | 256.74 | 170.00 | 112.85 | 1.35E-02 | 2.57E-02 |
| 8 | PC(14:0/18:2) | 774.5254 | 287.74 | 3582.98 | 4360.35 | 0.11 | 0.10 |
| 9 | PC(16:0/16:1) | 776.5478 | 392.35 | 774.49 | 944.72 | 5.92E-02 | 4.06E-02 |
| 10 | PC(16:0/16:0) | 778.5564 | 363.39 | 10094.11 | 8855.64 | 0.10 | 0.10 |
| 11 | PC(15:0/18:3) | 786.5243 | 280.77 | 107.03 | 89.66 | 0.20 | 0.17 |
| 12 | PC(15:0/18:2) | 788.5397 | 311.41 | 1546.56 | 1950.71 | 7.15E-03 | 1.92E-02 |
| 13 | PC(15:0/18:1) | 790.5554 | 346.79 | 1455.64 | 974.53 | 8.26E-03 | 2.07E-02 |
| 14 | PC(14:0/20:5) | 796.5096 | 248.98 | 200.35 | 208.58 | 0.74 | 0.42 |
| 15 | PC(14:0/20:4) | 798.5252 | 280.61 | 1402.49 | 1290.82 | 0.56 | 0.36 |
| 16 | PC(16:1/18:3) | 798.5238 | 263.17 | 312.25 | 295.24 | 0.64 | 0.39 |
| 17 | PC(16:0/18:3) | 800.5553 | 394.06 | 269.38 | 436.53 | 1.13E-04 | 2.27E-03 |
| 18 | PC(26:2/8:0) | 802.5550 | 473.51 | 522.14 | 591.42 | 0.59 | 0.37 |
| 19 | PC(16:0/18:2) | 802.5654 | 433.27 | 930.98 | 979.69 | 0.66 | 0.39 |
| 20 | PC(16:0/18:1) | 804.5624 | 334.04 | 25906.69 | 29510.92 | 5.49E-02 | 6.37E-02 |
| 21 | PC(18:0/16:0) | 806.5778 | 368.65 | 13251.60 | 10152.58 | 5.27E-03 | 1.62E-02 |
| 22 | PC(16:0/18:0) | 806.5904 | 499.77 | 140.06 | 117.01 | 6.60E-02 | 7.29E-02 |
| 23 | PC(20:5/15:0) | 810.5241 | 273.14 | 121.23 | 104.10 | 0.35 | 0.26 |
| 24 | PC(15:0/20:4) | 812.5283 | 388.64 | 55.07 | 42.68 | 5.81E-02 | 6.64E-02 |
| 25 | PC(17:0/18:2) | 816.5711 | 357.11 | 5810.28 | 7593.07 | 5.72E-04 | 4.36E-03 |
| 26 | PC(17:0/18:1) | 818.5866 | 390.67 | 2536.80 | 2087.97 | 6.81E-02 | 7.45E-02 |
| 27 | PC(17:0/18:0) | 820.6109 | 405.27 | 232.32 | 271.59 | 9.95E-02 | 9.62E-02 |
| 28 | PC(14:0/22:6) | 822.5243 | 269.47 | 594.49 | 667.00 | 0.30 | 0.23 |
| 29 | PC(16:1/20:5) | 822.5234 | 254.90 | 174.17 | 119.98 | 2.75E-02 | 4.11E-02 |
| 30 | PC(16:0/20:5) | 824.5411 | 297.10 | 22087.92 | 18949.11 | 0.32 | 0.24 |
| 31 | PC(16:1/20:4) | 824.5403 | 273.10 | 3293.54 | 3498.54 | 0.63 | 0.38 |
| 32 | PC(16:0/20:4) | 826.5568 | 327.87 | 76576.82 | 72744.63 | 0.62 | 0.38 |
| 33 | PC(18:2/18:2) | 826.5565 | 304.79 | 25110.02 | 37034.05 | 1.05E-03 | 5.81E-03 |
| 34 | PC(16:0/20:3) | 828.5618 | 304.92 | 2686.10 | 4173.25 | 1.42E-03 | 6.48E-03 |
| 35 | PC(18:0/18:2) | 830.5879 | 379.36 | 163154.58 | 176296.51 | 0.28 | 0.22 |
| 36 | PC(18:0/18:1) | 832.6138 | 479.46 | 363.14 | 350.67 | 0.74 | 0.42 |
| 37 | PC(22:6/15:0) | 836.5329 | 306.79 | 561.26 | 413.09 | 0.20 | 0.17 |
| 38 | PC(15:0/22:6) | 836.5401 | 292.71 | 512.71 | 734.49 | 4.00E-04 | 3.63E-03 |
| 39 | PC(17:0/20:5) | 838.5456 | 292.25 | 99.90 | 126.39 | 6.19E-02 | 1.78E-02 |
| 40 | PC(17:0/20:4) | 840.5709 | 350.64 | 2040.32 | 2444.92 | 4.54E-02 | 5.64E-02 |
| 41 | PC(17:0/20:3) | 842.5858 | 366.18 | 967.08 | 955.46 | 0.92 | 0.48 |
| 42 | PC(19:0/18:2) | 844.6015 | 401.55 | 1200.51 | 1378.44 | 9.30E-02 | 9.22E-02 |
| 43 | PC(19:0/18:1) | 846.6263 | 410.34 | 477.29 | 545.53 | 9.66E-02 | 9.45E-02 |
| 44 | PC(16:1/22:6) | 848.5395 | 276.57 | 1389.09 | 1444.44 | 0.77 | 0.43 |
| 45 | PC(16:0/22:6) | 850.5558 | 316.05 | 57560.48 | 66739.77 | 3.50E-02 | 4.75E-02 |
| 46 | PC(18:2/20:4) | 850.5565 | 297.44 | 9076.63 | 11530.32 | 2.83E-02 | 4.19E-02 |
| 47 | PC(18:1/20:4) | 852.5709 | 331.41 | 13268.59 | 13353.05 | 0.96 | 0.49 |
| 48 | PC(18:0/20:5) | 852.5717 | 344.48 | 11830.36 | 9044.54 | 2.67E-02 | 4.04E-02 |
| 49 | PC(18:0/20:4) | 854.5875 | 373.08 | 57760.34 | 60476.63 | 0.63 | 0.38 |
| 50 | PC(18:0/20:3) | 856.6022 | 391.69 | 36541.57 | 28218.05 | 4.03E-02 | 5.20E-02 |
| 51 | PC(18:0/20:2) | 858.6184 | 420.22 | 6103.35 | 4748.18 | 4.51E-02 | 5.61E-02 |
| 52 | PC(18:0/20:1) | 860.6344 | 452.92 | 518.40 | 347.20 | 1.96E-03 | 3.28E-02 |
| 53 | PC(22:6/17:0) | 864.5683 | 339.21 | 529.87 | 647.27 | 0.12 | 0.11 |
| 54 | PC(17:0/22:5) | 866.5877 | 345.44 | 607.42 | 627.51 | 0.69 | 0.41 |
| 55 | PC(19:0/20:4) | 868.6016 | 395.13 | 411.12 | 479.23 | 9.32E-02 | 9.23E-02 |
| 56 | PC(19:0/20:3) | 870.6166 | 414.08 | 141.86 | 123.29 | 0.36 | 0.26 |
| 57 | PC(20:5/20:4) | 872.5387 | 257.42 | 113.74 | 103.25 | 0.43 | 0.30 |
| 58 | PC(26:2/13:0) | 872.6436 | 410.49 | 240.06 | 227.89 | 0.66 | 0.40 |
| 59 | PC(22:6/18:2) | 874.5652 | 368.09 | 343.23 | 302.29 | 7.14E-02 | 7.71E-02 |
| 60 | PC(20:3/20:4) | 876.5634 | 287.27 | 300.11 | 350.80 | 0.11 | 0.10 |
| 61 | PC(18:1/22:6) | 876.5711 | 320.60 | 4078.03 | 4084.22 | 0.99 | 0.49 |
| 62 | PC(18:0/22:6) | 878.5878 | 361.44 | 21841.19 | 20693.65 | 0.49 | 0.33 |
| 63 | PC(18:0/22:5) | 880.5946 | 361.09 | 3775.85 | 3660.31 | 0.69 | 0.41 |
| 64 | PC(20:1/20:3) | 882.6013 | 360.85 | 242.84 | 235.42 | 0.64 | 0.39 |
| 65 | PC(18:0/22:4) | 882.6166 | 418.05 | 653.71 | 652.32 | 0.98 | 0.49 |
| 66 | PC(22:1/18:2) | 884.6233 | 404.32 | 502.72 | 445.59 | 9.38E-02 | 2.21E-02 |
| 67 | PC(22:1/18:1) | 886.6485 | 463.97 | 342.73 | 351.95 | 0.81 | 0.44 |
| 68 | PC(22:0/18:1) | 888.6631 | 492.95 | 155.05 | 139.53 | 0.30 | 0.23 |
| 69 | PC(24:4/17:2) | 892.6127 | 387.56 | 128.19 | 167.95 | 3.78E-03 | 1.30E-02 |
| 70 | PC(22:6/20:5) | 896.5381 | 245.50 | 18.13 | 19.55 | 0.67 | 0.40 |
| 71 | PC(22:5/20:4) | 900.5603 | 276.48 | 74.62 | 90.12 | 0.30 | 0.23 |
| 72 | PC(20:4/22:4) | 902.5857 | 326.08 | 159.63 | 165.79 | 0.70 | 0.41 |
| 73 | PC(20:2/22:6) | 902.5953 | 414.18 | 279.93 | 226.70 | 3.46E-02 | 4.72E-02 |
| 74 | PC(22:6/20:1) | 904.6011 | 363.66 | 256.54 | 223.90 | 0.18 | 0.15 |
| 75 | PC(20:4/22:1) | 908.6322 | 416.40 | 338.61 | 311.97 | 0.55 | 0.35 |
| 76 | PC(18:0/24:4) | 910.6452 | 437.83 | 184.49 | 174.66 | 0.61 | 0.38 |
| 77 | PC(22:0/20:4) | 910.6443 | 456.04 | 124.58 | 175.44 | 0.03 | 4.41E-02 |
| 78 | PC(26:1/16:1) | 914.6786 | 504.41 | 207.38 | 223.27 | 0.50 | 0.33 |
| 79 | PC(24:0/18:1) | 916.7178 | 626.04 | 185.51 | 157.40 | 0.18 | 0.15 |
| 80 | PC(22:6/21:0) | 920.6406 | 424.19 | 142.05 | 159.10 | 0.25 | 0.20 |
| 81 | PC(23:0/20:5) | 922.6566 | 454.45 | 904.67 | 782.35 | 0.12 | 0.11 |
| 82 | PC(22:5/22:6) | 924.5882 | 391.78 | 1457.70 | 1282.42 | 8.83E-02 | 2.14E-02 |
| 83 | PC(23:0/20:4) | 924.6615 | 454.32 | 177.55 | 156.40 | 0.17 | 0.15 |
| 84 | PC(24:1/20:4) | 936.6615 | 457.58 | 104.60 | 78.53 | 1.02E-02 | 2.30E-02 |
| 85 | PC(20:4/24:0) | 938.6787 | 497.00 | 119.34 | 123.08 | 0.74 | 0.42 |

**Figure S1 The formula to calculate the absolute concentration of PCs (RF_PC_=1.51)**
